# Geriatric syndromes, multimorbidity, and life satisfaction among older adults in India: a cross-sectional analysis of the Longitudinal Ageing Study in India (LASI)

**DOI:** 10.21203/rs.3.rs-9722448/v1

**Published:** 2026-05-29

**Authors:** Bhrigu Jain, Akash Jaiswal, Mujtaba Waris, Jeetendra Yadav, Pramod Kumar, Prasun Chatterjee

**Affiliations:** Medanta The Medicity; Fortis Memorial Research Institute; University Hospitals Sussex NHS Foundation Trust; Indian Council of Medical Research; All India Institute of Medical Sciences; Indraprastha Apollo Hospitals

**Keywords:** Aged, Multimorbidity, Geriatric Assessment, Personal Satisfaction, Health Surveys, India, LASI, Geriatric Syndromes

## Abstract

**Background.:**

Older adults in low- and middle-income countries are increasingly affected by both chronic-disease multimorbidity (MM) and geriatric syndromes (GS), but evidence on their joint impact on subjective well-being from the Indian subcontinent is limited. We examined the prevalence of MM and GS, and quantified their independent and joint associations with life satisfaction, in a nationally representative sample of older Indians.

**Methods.:**

We conducted a cross-sectional analysis of Wave 1 of the Longitudinal Ageing Study in India (LASI, 2017–18), restricted to community-dwelling adults aged 60 years or older (n = 30,811 with complete data on key variables; weighted mean age 68.6 years, SD 7.3). Multimorbidity was defined as two or more of nine self-reported physician-diagnosed chronic conditions. Geriatric syndromes were defined as the presence of at least one of seven syndromes: cognitive impairment (general cognitive factor score below the 10th weighted percentile), hearing impairment, vision impairment (poor self-rated near or distance vision), falls in the past 2 years, urinary incontinence, depression (Center for Epidemiologic Studies Depression-10 score ≥ 4 symptoms), and underweight (body-mass index < 18.5 kg/m^2^). Life satisfaction was measured using the 5-item Satisfaction with Life Scale (SWLS). All analyses applied LASI individual analysis weights. Associations were estimated using sequentially adjusted weighted linear regression with sandwich-estimator standard errors.

**Results.:**

The weighted prevalence of MM was 23.4 % and of any GS 70.5 %. State-level MM prevalence ranged from 4.1 % in Arunachal Pradesh to 55.7 % in Kerala. After full adjustment, the presence of any GS was associated with a 2.36-point lower life satisfaction score (95 % CI − 2.61 to − 2.11; p < 0.001), with a clear dose–response by GS count (1 GS: β = − 1.52; 2 GS: β = − 2.89; ≥ 3 GS: β = − 4.33; all p < 0.001). In contrast, MM was not independently associated with life satisfaction in the adjusted model (β = + 0.08; 95 % CI − 0.33 to + 0.49; p = 0.71), nor was the MM × GS interaction term (β = − 0.19; 95 % CI − 0.69 to + 0.30; p = 0.44). Higher education, greater wealth, co-residence with family, urban residence, and female sex were independently associated with higher life satisfaction. Findings were robust to a sensitivity analysis excluding depression from the GS composite (GS β = − 1.48; 95 % CI − 1.72 to − 1.23).

**Conclusions.:**

In community-dwelling older Indians, geriatric syndromes, not multimorbidity are the principal correlate of reduced life satisfaction, with a graded dose–response by number of syndromes. Primary-care programmes for older adults in India, including the National Programme for Health Care of the Elderly and the Ayushman Bharat Health and Wellness Centres, should prioritise structured screening and management of geriatric syndromes alongside chronic-disease care, particularly in rural and lower-wealth populations where GS prevalence is highest but specialist access is most limited.

**Trial registration:**

Not applicable. This study is a secondary analysis of observational survey data and did not involve a health-care intervention.

## Background

India is undergoing a rapid demographic transition. Adults aged 60 years and above currently account for approximately 10% of the Indian population and are projected to exceed 20% by 2050, adding nearly 200 million older adults in a single generation [1, 2]. This transition has unfolded against the backdrop of a health system historically designed around single-disease, organ-specific care, an approach increasingly inadequate for older patients whose presentations are dominated by chronic disease clustering, functional decline, and atypical, multi-system problems [3, 4].

Two complementary frameworks have evolved to describe health complexity in later life. Multimorbidity (MM), defined as the co-existence of two or more chronic conditions in a single individual, reflects the accumulation of organ-based diseases, with global prevalence estimates in older populations ranging from 37% to 56% [5, 6]. Geriatric syndromes (GS) — including falls, cognitive impairment, urinary incontinence, malnutrition, depression, and sensory deficits — represent multifactorial health states that do not map cleanly onto any single disease category and typically arise from shared underlying contributors such as inflammaging, sarcopenia, and loss of physiological reserve [7, 8]. The two frameworks are related but distinct: MM captures disease burden, whereas GS captures clinical vulnerability and functional impact.

The combined impact of MM and GS is increasingly recognised in high-income settings but remains poorly quantified in low- and middle-income countries, where the burden is compounded by weaker primary-care infrastructure, a shortage of trained geriatric workforce, and limited insurance coverage [9, 10]. In India, the National Programme for Health Care of the Elderly (NPHCE) was launched in 2010 to strengthen geriatric care delivery, and more recently the Ayushman Bharat Pradhan Mantri Jan Arogya Yojana and the Health and Wellness Centres have expanded coverage for chronic-disease screening [11]. However, case-finding for geriatric syndromes at the primary-care level remains inconsistent, and empirical evidence to inform integrated screening policies is limited.

Life satisfaction is a widely used proxy for overall quality of life that captures the subjective well-being dimension of health, integrating physical function, psychological state, and social context into a single self-reported measure [12]. Higher life satisfaction is prospectively associated with lower all-cause mortality, better health behaviours, and reduced healthcare utilisation, independent of objectively measured health status [13]. While prior work has examined the impact of MM on life satisfaction [14, 15], and a smaller body of literature has explored GS and life satisfaction [16], to our knowledge no study from the Indian subcontinent has evaluated their joint effect in a nationally representative sample using design-appropriate weighted analyses.

The Longitudinal Ageing Study in India (LASI) provides a unique opportunity to address this gap. LASI Wave 1 enrolled 73,408 adults aged 45 years or older across 35 states and union territories using a multistage stratified probability design, capturing detailed information on chronic diseases, geriatric syndromes, and psychosocial well-being [17]. Using the harmonised version of LASI Wave 1 [18], we examined: (i) the weighted prevalence and sociodemographic correlates of MM and GS in community-dwelling older Indians; (ii) the independent and joint associations of MM and GS with life satisfaction, adjusting for sociodemographic covariates; and (iii) the dose–response between number of geriatric syndromes and life satisfaction.

## Methods

### Study design and data source

We conducted a cross-sectional analysis of LASI Wave 1, conducted between April 2017 and December 2018 by the International Institute for Population Sciences in collaboration with the Harvard T. H. Chan School of Public Health, the University of Southern California, and the Ministry of Health and Family Welfare, Government of India [17]. LASI used a multistage stratified area probability cluster sampling design: a three-stage design in rural areas (primary sampling unit [PSU] → village → household) and a four-stage design in urban areas (PSU → ward → census enumeration block → household). A total of 73,408 adults aged 45 years or older and their spouses were interviewed across 35 states and union territories. The present analysis used the Harmonized LASI dataset (Version A.3, released by the Gateway to Global Aging Data, University of Southern California) [18], which provides cleaned and standardised variables suitable for international comparative ageing research. Reporting follows the Strengthening the Reporting of Observational Studies in Epidemiology (STROBE) checklist for cross-sectional studies (provided as Additional file 1).

### Study population

**Figure 1:**
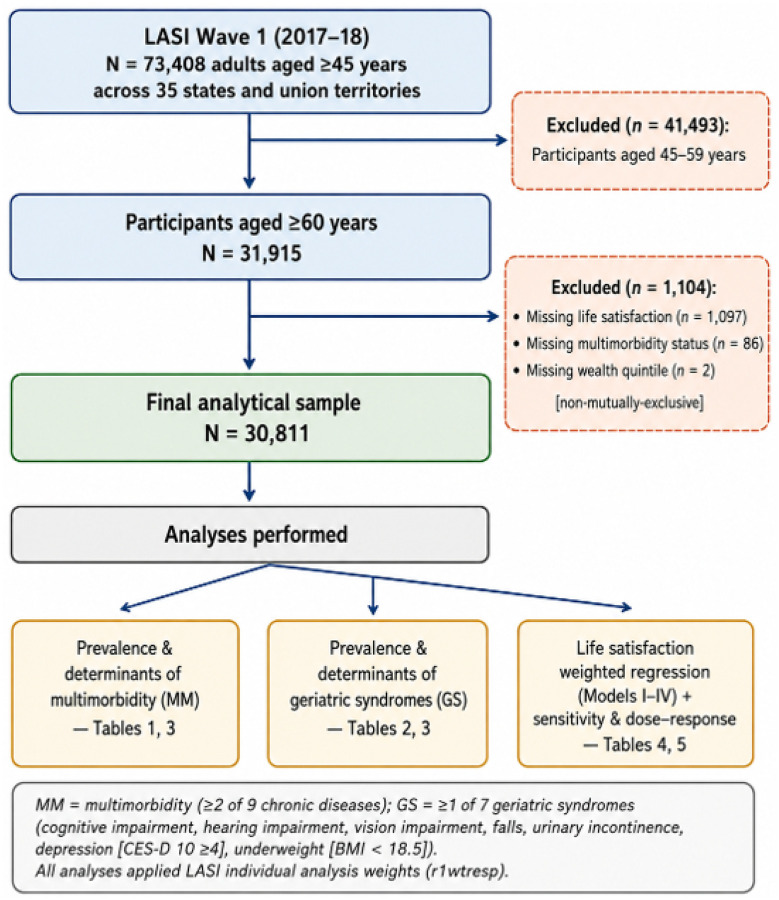
Participant flow diagram. STROBE-style derivation of the analytical sample from the Longitudinal Ageing Study in India (LASI) Wave 1. MM = multimorbidity (≥ 2 of 9 chronic diseases); GS = geriatric syndromes (cognitive, hearing, and vision impairment; falls; urinary incontinence; depression [CES-D 10 ≥ 4]; and underweight [BMI < 18.5]). Exclusions for missing data on key analysis variables (multimorbidity, life satisfaction, or wealth quintile) are non-mutually exclusive; the total of 1,104 is unique participants with at least one missing variable.

We restricted the sample to participants aged 60 years or older (n = 31,915). Participants with missing data on any of the key analysis variables of life satisfaction, multimorbidity status, or wealth quintile were excluded, yielding a final analytical sample of n = 30,811. Missing values were concentrated in life satisfaction (n = 1,097, 3.4%), multimorbidity status (n = 86, 0.3%), and wealth quintile (n = 2). Other key covariates had no missing values in the 60 + subset (Fig. 1).

### Variables

#### Outcome: life satisfaction

Life satisfaction was measured using the Satisfaction with Life Scale (SWLS) [12]. Participants rated their agreement with five statements (‘In most ways, my life is close to ideal’; ‘The conditions of my life are excellent’; ‘I am satisfied with my life’; ‘So far, I have got the important things I want in life’; and ‘If I could live my life over, I would change almost nothing’) on a seven-point Likert scale (1 = strongly disagree to 7 = strongly agree). The composite score was computed as the sum of the five items, ranging from 5 to 35. Life satisfaction was analysed both as a continuous variable in regression models and as a categorical variable for descriptive purposes (Low: 5–20; Medium: 21–25; High: 26–35), following the categorisation used in the LASI India Report [17].

#### Exposure 1: multimorbidity

Multimorbidity (MM) was defined as the presence of two or more self-reported physician-diagnosed chronic conditions among nine assessed in LASI: hypertension, chronic heart disease, stroke, chronic lung disease, diabetes mellitus, any cancer or malignant tumour, any bone or joint disease, any neurological or psychiatric disease, and high cholesterol. MM was modelled as a binary indicator (yes / no).

#### Exposure 2: geriatric syndromes

Seven geriatric syndromes were operationalised from LASI variables. (1) Cognitive impairment was defined as a general cognitive factor score (derived from a comprehensive cognitive battery including word recall, orientation, serial 7s, and executive function tasks) falling below the 10th weighted percentile of the study-population score distribution. (2) Hearing impairment was defined as self-reported lifetime hearing or ear-related condition. (3) Vision impairment was defined as self-rated near or distance eyesight as ‘poor’ or ‘very poor’. (4) Falls were ascertained by self-report of any fall in the past 2 years. (5) Urinary incontinence was based on self-reported lifetime diagnosis. (6) Depression was defined as a Center for Epidemiologic Studies Depression-10 (CES-D 10) score indicating four or more depressive symptoms, the standard LASI population-level depression measure [17]. (7) Underweight was defined as measured body-mass index < 18.5 kg/m^2^ (Asian underweight cut-off) [19]. We modelled GS three ways: as a binary indicator (any GS vs none); as a count (0, 1, 2, ≥ 3); and individually.

We chose the CES-D 10 over the Composite International Diagnostic Interview – Short Form (CIDI-SF) for the depression component because the CIDI-SF was administered to only a subset of LASI participants (~ 6%), whereas the CES-D 10 covers more than 97% of the sample and is the standard depression instrument in LASI population analyses.

### Covariates

Sociodemographic covariates were selected a priori based on prior LASI literature on life satisfaction [15]: age group (60–69, 70–79, ≥ 80 years); sex (male, female); living arrangement (living alone; with spouse only; with spouse and children/others; with children/others without spouse); education (less than upper secondary; upper secondary; tertiary, using the harmonised LASI three-category education variable); wealth quintile based on weighted per-capita household consumption expenditure (poorest, poorer, middle, richer, richest); place of residence (rural; urban); and region (North; Central; East; Northeast; West; South), grouped from the LASI interview-state variable using the standard six-region scheme of the LASI India Report [17].

### Statistical analysis

All analyses applied the LASI individual analysis weights (variable r1wtresp), which are post-stratified to the 2011 Census of India age-sex-state distribution. Weighted prevalences are reported as percentages. Continuous variables (life satisfaction score) are summarised as weighted mean ± standard deviation (SD). Bivariate associations were tested with Pearson chi-square tests. The associations of MM, GS, and their joint occurrence with life satisfaction were modelled using sequentially adjusted weighted ordinary least-squares linear regression with sandwich-estimator (Huber–White) standard errors to account for design-induced heteroskedasticity. Four models were specified: Model I (each exposure alone, bivariate); Model II (MM, GS, and MM × GS interaction term, mutually adjusted); Model III (sociodemographic covariates only); Model IV (full adjustment for both exposures and all covariates).

Two pre-specified secondary analyses were performed. First, we examined the dose–response between number of geriatric syndromes (0, 1, 2, ≥ 3) and life satisfaction in a fully adjusted model. Second, we conducted a sensitivity analysis re-fitting Model IV with the GS composite re-derived to exclude depression, given the conceptual overlap between the depression construct and the SWLS instrument, to test whether the GS effect persisted without the depression component. All analyses were performed in Stata version 17 (StataCorp. 2021. Stata: Release 17. Statistical Software. College Station, TX: StataCorp LLC.). Statistical significance was set at a two-sided α = 0.05.

## Results

### Sample characteristics

The final analytical sample comprised 30,811 adults aged 60 years or older (Fig. 1), with a weighted mean age of 68.6 years (standard deviation [SD] 7.3) and an age range of 60 to 116 years. The mean life satisfaction score was 23.95 (SD 7.23, range 5–35). Sociodemographic characteristics are shown in Table 1: 50.9% were female; 62.5% were aged 60–69 years, 27.7% aged 70–79 years, and 9.8% aged ≥ 80 years; 69.4% resided in rural areas; and 80.5% had less than upper-secondary education.

### Prevalence and determinants of multimorbidity

The weighted prevalence of multimorbidity was 23.4%, and 51.7% of older adults reported at least one chronic condition. MM prevalence was higher in the 70–79 age group (25.2%) than at 60–69 years (22.8%) or ≥ 80 years (21.9%) (p < 0.001), the dip at the oldest age consistent with survivor selection. MM prevalence was higher in females than males (24.5% vs 22.2%; p < 0.001), in urban than rural residents (36.0% vs 17.8%; p < 0.001), and rose monotonically across wealth quintiles from 12.9% in the poorest to 36.5% in the richest (p < 0.001). Educational attainment showed a similar gradient (20.2% in those with less than upper-secondary education vs 40.6% in those with tertiary education; p < 0.001). State-level prevalence ranged from 4.1% in Arunachal Pradesh to 55.7% in Kerala (Fig. 3).

### Prevalence and determinants of geriatric syndromes

At least one GS was present in 70.5% of older adults (Table 2). Prevalence rose with age (66.4% at 60–69; 74.3% at 70–79; 85.5% at ≥ 80; p < 0.001) and was higher in females than males (73.9% vs 66.9%; p < 0.001). In contrast to MM, GS prevalence was inversely related to socioeconomic status: 74.7% in those with less than upper-secondary education vs 45.3% in those with tertiary education; 76.7% in the poorest wealth quintile vs 63.9% in the richest; and 74.8% in rural vs 60.5% in urban areas (all p < 0.001). The most common individual syndromes were depression (30.6%), vision impairment (27.6%), underweight (26.1%), and falls in the past 2 years (22.2%) (Table 3).

### Co-occurrence of multimorbidity and geriatric syndromes

Five of the seven geriatric syndromes were significantly more prevalent among participants with MM than among those without (Table 3): hearing impairment (12.4% vs 8.0%; p < 0.001), falls (25.7% vs 21.1%; p < 0.001), urinary incontinence (6.6% vs 3.2%; p < 0.001), and depression (34.3% vs 29.4%; p < 0.001). Conversely, two syndromes were less prevalent in the MM group: cognitive impairment (7.9% vs 10.5%; p < 0.001) and underweight (11.8% vs 30.4%; p < 0.001). Vision impairment did not differ significantly by MM status.

### Life satisfaction across MM and GS groups

Mean life satisfaction was substantially lower in those with any GS than in those without (22.65 vs 25.66; p < 0.001) but did not differ meaningfully by MM status alone (23.97 with vs 23.41 without; Table 4). When stratified into four MM × GS groups, life satisfaction was highest in the ‘MM only’ group (mean 26.21) — even higher than in the ‘Neither’ group (25.49) — and lowest in the ‘GS only’ (22.55) and ‘Both’ (22.98) groups (Fig. 4a). The proportion of participants in the ‘High’ life-satisfaction category was 61.5% in the MM-only group, 54.6% in the Neither group, and only 38.0% in the GS-only group.

A clear dose–response relationship was observed between number of geriatric syndromes and life satisfaction (Fig. 4b): mean scores fell from 25.66 (0 syndromes) to 23.76 (1 syndrome), 22.26 (2 syndromes), and 20.63 (≥ 3 syndromes).

### Multivariable regression analyses

**Figure 2:**
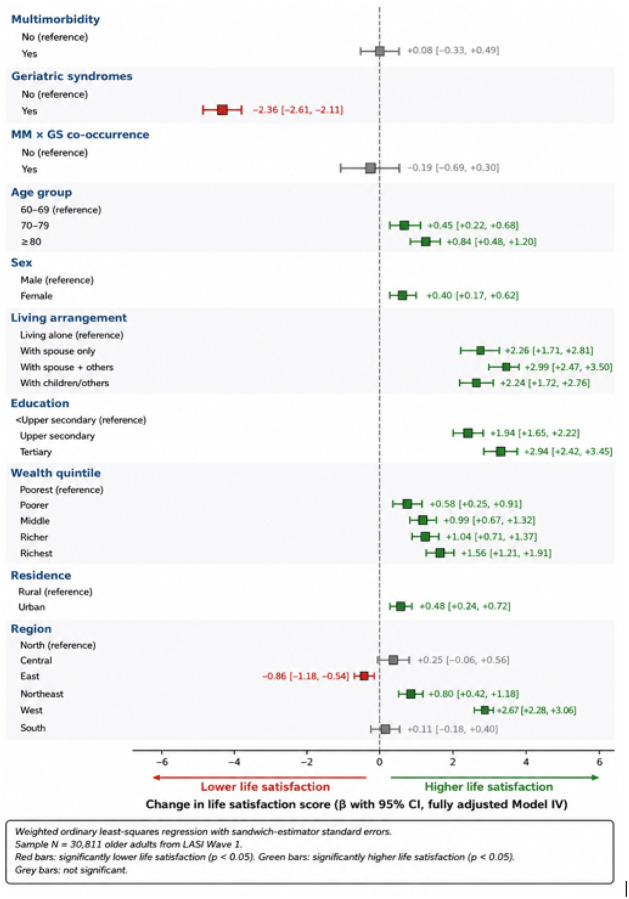
Forest plot of adjusted associations with life satisfaction (Model IV, fully adjusted). Squares show point estimates of the change in SWLS score (β); horizontal bars show 95 % confidence intervals. Estimates are from weighted ordinary least-squares regression with sandwich-estimator standard errors (N = 30,811). Red bars indicate significantly lower life satisfaction (p < 0.05); green bars significantly higher; grey bars not statistically significant.

Sequentially adjusted regression estimates are presented in Table 5 and visualised in Fig. 2. In the bivariate Model I, MM was associated with slightly higher life satisfaction (β = + 0.56; 95% CI + 0.32 to + 0.81), GS with substantially lower life satisfaction (β = − 3.01; 95% CI − 3.23 to − 2.80), and the joint occurrence of MM and GS with lower life satisfaction (β = − 0.67; 95% CI − 0.95 to − 0.39).

In Model II, mutually adjusting for MM, GS, and their interaction, the GS effect remained large and statistically significant (β = − 2.94), the MM main effect strengthened (β = + 0.72), and the interaction term attenuated to non-significance (β = − 0.29; 95% CI − 0.80 to + 0.21; p = 0.26).

In the fully adjusted Model IV, GS remained the dominant predictor of lower life satisfaction (β = − 2.36; 95% CI − 2.61 to − 2.11; p < 0.001), whereas MM was no longer associated with life satisfaction (β = + 0.08; 95% CI − 0.33 to + 0.49; p = 0.71), and the MM × GS interaction was non-significant (β = − 0.19; 95% CI − 0.69 to + 0.30; p = 0.44). Higher life satisfaction was independently associated with older age (≥ 80 years vs 60–69: β = + 0.84), female sex (β = + 0.40), co-residence with family (with spouse and children: β = + 2.99 vs living alone), higher education (tertiary: β = + 2.94), greater household wealth (richest vs poorest quintile: β = + 1.56), and urban residence (β = + 0.48). Substantial regional variation was observed, with the West region showing the highest scores (β = + 2.67 vs North) and the East the lowest (β = − 0.86).

### Dose–response and sensitivity analyses

In the fully adjusted dose–response model, life satisfaction declined progressively with each additional geriatric syndrome relative to participants with none (1 GS: β = − 1.52, 95% CI − 1.77 to − 1.28; 2 GS: β = − 2.89, 95% CI − 3.18 to − 2.60; ≥ 3 GS: β = − 4.33, 95% CI − 4.67 to − 3.98; all p < 0.001). The decrement associated with three or more syndromes (approximately 4.3 SWLS points) is approximately one-fifth of the scale’s interquartile range, representing a clinically meaningful effect.

In the sensitivity analysis re-fitting Model IV with depression excluded from the GS composite, the GS effect remained substantial (β = − 1.48; 95% CI − 1.72 to − 1.23; p < 0.001), confirming that the association between GS and life satisfaction is not driven solely by construct overlap between depression and the SWLS.

## Discussion

### Main findings

In this nationally representative weighted analysis of 30,811 community-dwelling older Indians, we found that geriatric syndromes, not multimorbidity, are the principal correlate of reduced life satisfaction. After full adjustment for sociodemographic covariates, the presence of any GS was associated with a 2.4-point decrement in life satisfaction with a clear dose–response by number of syndromes, whereas multimorbidity had no independent association and the MM × GS interaction was non-significant. To our knowledge, this is the first weighted analysis from the Indian subcontinent to jointly quantify the contributions of MM and GS to life satisfaction in a nationally representative sample of older adults.

### MM, healthcare access, and the diagnostic-detection paradox

**Figure 3:**
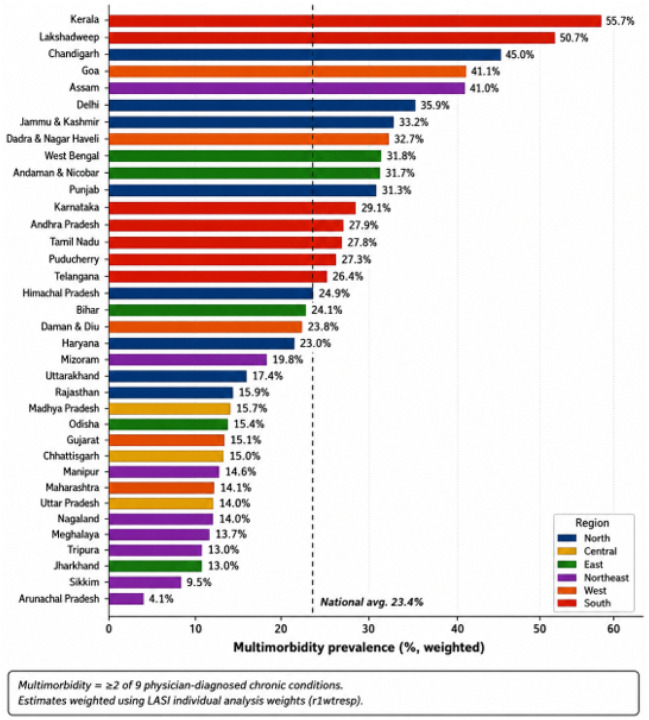
State-wise weighted prevalence of multimorbidity among older adults (≥ 60 years) in India, LASI Wave 1 (2017–18). The dashed vertical line indicates the national weighted average (23.4 %). Bars are colour-coded by the LASI six-region scheme. The Sikkim estimate (n = 38) reflects the limited Sikkim representation in the harmonised dataset and should be interpreted with caution; Sikkim was not part of the LASI Wave 1 sampling frame.

**Figure 4:**
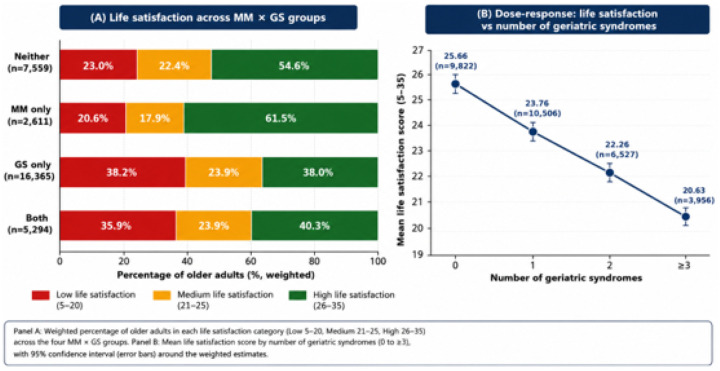
Life satisfaction by multimorbidity and geriatric syndrome status. (a) Weighted distribution of life satisfaction categories (Low: 5–20; Medium: 21–25; High: 26–35) across the four MM × GS groups (neither; MM only; GS only; both). (b) Dose–response between number of geriatric syndromes (0, 1, 2, ≥ 3) and mean life satisfaction score, with 95 % confidence intervals.

Our overall MM prevalence of 23.4% is at the lower end of international estimates; the recent global meta-analysis by Chowdhury and colleagues reported a pooled prevalence of 37% in older adults [20]. Two findings, however, suggest that the apparently lower MM burden in India largely reflects diagnostic detection rather than true biology. First, MM prevalence rose monotonically with both wealth (12.9% in the poorest vs 36.5% in the richest quintile) and education (20.2% in those with less than upper-secondary schooling vs 40.6% in tertiary-educated participants) and was almost twice as high in urban as rural residents, gradients consistent with differential healthcare contact and case-finding rather than excess disease in the wealthy. Second, the striking state-level variation (Fig. 3), from 4.1% in Arunachal Pradesh to 55.7% in Kerala, is unlikely to reflect true tenfold biological differences and instead mirrors interstate gradients in health-system maturity and screening capacity [21].

This interpretation is reinforced by the apparently paradoxical finding that life satisfaction was, if anything, higher in the ‘MM only’ group than in the ‘Neither’ group (26.21 vs 25.49). After adjustment, the MM main effect collapsed to null. The most parsimonious explanation is that, in the Indian context, having a diagnosed chronic condition is itself a marker of healthcare engagement, education, and economic resources, all of which are independently associated with higher life satisfaction. Once these structural determinants are controlled for, MM itself does not carry an additional subjective penalty. This finding aligns with the diagnostic-access interpretation of Pati and colleagues [22] in Odisha and warrants a reframing of what ‘high MM prevalence’ actually represents in Indian population data.

### Geriatric syndromes: the under-recognised burden

In striking contrast to MM, the prevalence of geriatric syndromes was high (70.5%) and showed an inverse socioeconomic gradient: more prevalent in rural residents, in those without formal education, and in lower wealth quintiles. Most importantly, GS was the dominant adjusted predictor of life satisfaction, with a robust dose–response by number of syndromes that survived sensitivity analysis excluding depression. Three older adults in ten in our sample had two or more concurrent geriatric syndromes, and these individuals scored 2.9 SWLS points lower than those with none, a difference comparable in magnitude to the gap between tertiary-educated and unschooled participants.

The contrasting socioeconomic patterns of MM and GS are clinically meaningful. MM is partly a diagnostic construct, cases must be detected to be counted, whereas many geriatric syndromes (falls, incontinence, underweight) can be ascertained through direct inquiry and measurement and are therefore less sensitive to health-system contact. The inverse GS gradient is likely to reflect genuine excess burden in disadvantaged populations, compounded by limited access to preventive services, rehabilitation, and assistive devices. Reliance on MM alone as a summary measure of health complexity will therefore systematically underestimate the health burden borne by older adults in India’s poorest and most rural communities.

### Co-occurrence of MM and individual geriatric syndromes

Participants with MM had higher prevalence of hearing impairment, falls, urinary incontinence, and depression, consistent with prior cohort evidence from the Irish Longitudinal Study on Ageing [23] and the English Longitudinal Study of Ageing [24]. Plausible mechanisms include shared upstream risk factors (age, vascular disease, inflammaging); polypharmacy as a downstream mediator that increases falls, incontinence, and cognitive disturbance; and progression along a frailty continuum in which accumulating chronic-disease burden erodes physiological reserve and precipitates geriatric syndromes [7, 25]. The clinical implication is that primary-care visits for chronic-disease management should incorporate brief geriatric screening, for example, a one-question fall screen, the three-item incontinence query, and the two-item depression screen (PHQ-2), as these syndromes are unlikely to be volunteered by patients but are common and largely modifiable.

### Paradoxical findings

Two findings deserve specific interpretation. First, cognitive impairment and underweight were less prevalent among participants with MM, the opposite of what one might expect. For cognitive impairment, this almost certainly reflects detection: older adults with MM are more likely to engage with healthcare, to have higher education, and to score better on cognitive testing — which uses an unweighted population-relative threshold (below the 10th percentile of the score distribution). For underweight, the lower prevalence in MM reflects the higher BMI of the diagnosed-chronic-disease population: hypertension, diabetes, and high cholesterol, three of the four most common conditions in the MM definition, are themselves associated with higher BMI.

Second, in the fully adjusted model, female sex was associated with higher life satisfaction (β = + 0.40) despite women having higher prevalence of both MM and GS. This pattern, higher objective morbidity coexisting with higher subjective well-being in women, has been reported internationally and attributed to differences in coping strategies, social-network embeddedness, and normative reference points against which self-assessment is made [14]. An explanation specific to the Indian context is that older women in multigenerational households may derive meaning and status from caregiving and grandparent roles that partially offset the subjective impact of physical morbidity. Importantly, this finding emerged only after adjustment; in unadjusted comparisons, women had lower life satisfaction, and the direction reversed once living arrangement, education, and wealth, all of which differ systematically by sex in India and were controlled for.

### Policy and clinical implications

Three implications follow directly. First, brief structured screening for the geriatric syndromes most strongly associated with reduced well-being (falls, incontinence, depression, hearing impairment, and undernutrition) should be embedded into every chronic-disease follow-up visit for older adults under NPHCE and at Ayushman Bharat Health and Wellness Centres. Second, task-sharing models using community health workers (Accredited Social Health Activist (ASHA) and Auxiliary Nurse Midwife (ANM) cadres) for first-level geriatric screening can expand reach in rural and low-wealth settings where GS prevalence is highest, and specialist access is most limited. Third, the dose–response by number of syndromes argues for a stratified care model: older adults with three or more syndromes (approximately 14% of our sample) represent a high-priority population for comprehensive geriatric assessment and multidisciplinary intervention.

### Strengths and limitations

Strengths of this study include the use of the largest and most comprehensive survey of ageing ever conducted in India, with rigorous multistage stratified sampling across 35 states and union territories; appropriate application of LASI individual analysis weights to all descriptive and regression estimates; the use of validated multi-item instruments (SWLS, CES-D 10) and objectively measured anthropometry; the use of a broad cognitive factor score rather than a single test; and pre-specified sensitivity and dose–response analyses to test the robustness of our principal finding.

Several limitations warrant acknowledgement. First, the design is cross-sectional and we cannot infer causality; reverse causation is plausible whereby low life satisfaction influences health behaviours that promote development of GS, and longitudinal analyses in subsequent LASI waves will be needed to clarify temporal ordering. Second, MM was based on self-reported physician diagnoses, which systematically under-ascertain disease in populations with limited healthcare access, explicitly acknowledged in our interpretation above. Third, several GS components were self-reported (hearing, urinary incontinence, vision) without validated instruments. Fourth, our cognitive-impairment threshold was population-relative rather than clinically validated, capturing relative rather than absolute decline. Fifth, residual confounding by unmeasured factors, including caregiver burden, social engagement, religiosity, and chronic pain is possible. Sixth, while the harmonised LASI dataset includes Sikkim records, the LASI India Report indicates that Sikkim was excluded from the Wave 1 sampling frame, and our state-level estimate for Sikkim should be interpreted with caution. Seventh, we did not adjust for the complex multistage cluster design beyond applying individual analysis weights and sandwich standard errors; full Taylor-series linearisation with stratum and PSU identifiers might yield slightly different precision estimates.

### Future research directions

Longitudinal analyses using LASI Waves 2 and 3 will allow us to test the temporal ordering of MM, GS, and life satisfaction; to identify which specific GS combinations carry the greatest well-being penalty; and to evaluate whether intervening on modifiable GS components (depression treatment, fall prevention, undernutrition) improves life satisfaction. Implementation research is needed to test whether embedding brief geriatric screening into NPHCE-linked primary-care visits improves case detection and downstream outcomes. Finally, qualitative work is needed to understand why older Indian women despite higher objective morbidity report higher life satisfaction after adjustment, as the answer has implications for the cultural validity of subjective well-being instruments in South Asian populations.

## Conclusions

Geriatric syndromes are highly prevalent (70%) and disproportionately affect rural and lower-wealth older adults in India, and they are the dominant adjusted correlate of reduced life satisfaction with a clear dose–response by number of syndromes. Multimorbidity, in contrast, is largely a marker of diagnostic access and does not independently predict life satisfaction once geriatric syndromes and sociodemographic factors are controlled for. Primary-care programmes for older adults in India should prioritise structured screening and management of geriatric syndromes particularly depression, falls, sensory impairments, and undernutrition, alongside chronic-disease care, with targeted attention to rural and low-wealth populations where GS burden is highest, and specialist access is most limited.

## Supplementary Material

Supplementary Files

This is a list of supplementary files associated with this preprint. Click to download.

- Additionalfile1STROBEchecklist.docx

## Figures and Tables

**Table 1:** Sociodemographic characteristics and weighted prevalence of multimorbidity

Variable	Category	N	Any morbidity %	MM %	P any	P mm
**Mean age (years), mean (SD)**	68.6 (7.3)					
Overall		30,811	51.7	23.4		
Age group					< 0.001	< 0.001
	60–69	18,763 (62.5)	49.8	22.8		
	70–79	8,907 (27.7)	55.7	25.2		
	80+	3,141 (9.8)	52.1	21.9		
Sex					< 0.001	< 0.001
	Male	14,789 (49.1)	48.9	22.2		
	Female	16,022 (50.9)	54.4	24.5		
Living arrangement					< 0.001	0.001
	Living alone	1,581 (5.4)	50.8	20.5		
	With spouse only	4,642 (16.1)	52.1	24.8		
	With spouse and children/others	15,099 (48.0)	50.8	23.0		
	With children/others (no spouse)	9,489 (30.5)	53.1	23.8		
Education					< 0.001	< 0.001
	Less than upper sec	23,949 (80.5)	48.6	20.2		
	Upper secondary	5,583 (15.6)	62.4	35.6		
	Tertiary	1,279 (3.9)	71.7	40.6		
Wealth quintile					< 0.001	< 0.001
	Poorest	5,553 (19.9)	37.4	12.9		
	Poorer	5,684 (20.1)	46.5	18.0		
	Middle	6,164 (20.1)	52.5	22.4		
	Richer	6,424 (20.0)	56.3	27.1		
	Richest	6,986 (20.0)	65.8	36.5		
Place of residence					< 0.001	< 0.001
	Rural	20,383 (69.4)	45.6	17.8		
	Urban	10,428 (30.6)	65.4	36.0		
Region					< 0.001	< 0.001
	North	5,654 (13.6)	55.0	23.6		
	Central	4,584 (24.1)	39.3	14.6		
	East	4,761 (13.7)	51.8	24.0		
	Northeast	4,219 (3.0)	44.6	14.9		
	West	3,660 (10.2)	45.2	19.4		
	South	7,933 (35.5)	61.2	30.9		

Sociodemographic characteristics of the analytical sample (N = 30,811) and weighted prevalence of any chronic morbidity (≥ 1 of 9 conditions) and multimorbidity (MM; ≥ 2 of 9 conditions). N column shows unweighted counts with weighted percentage distribution in parentheses. Prevalence columns show weighted percentages of the row subgroup with the outcome. p-values from Pearson chi-square tests on the unweighted counts (design-adjusted Rao-Scott statistics were not computed). All estimates use the LASI individual analysis weight r1wtresp.

**Table 2:** Weighted prevalence of any geriatric syndrome by sociodemographic characteristics

Variable	Category	N	GS prevalence %	P value
Overall		30,811	70.5	
Age group				< 0.001
	60–69	18,763	66.4	
	70–79	8,907	74.3	
	80+	3,141	85.5	
Sex				< 0.001
	Male	14,789	66.9	
	Female	16,022	73.9	
Living arrangement				< 0.001
	Living alone	1,581	80.3	
	With spouse only	4,642	68.1	
	With spouse and children/others	15,099	66.1	
	With children/others (no spouse)	9,489	76.9	
Education				< 0.001
	Less than upper sec	23,949	74.7	
	Upper secondary	5,583	54.8	
	Tertiary	1,279	45.3	
Wealth quintile				< 0.001
	Poorest	5,553	76.7	
	Poorer	5,684	73.4	
	Middle	6,164	71.4	
	Richer	6,424	67.0	
	Richest	6,986	63.9	
Place of residence				< 0.001
	Rural	20,383	74.8	
	Urban	10,428	60.5	
Region				< 0.001
	North	5,654	67.5	
	Central	4,584	75.7	
	East	4,761	79.6	
	Northeast	4,219	68.7	
	West	3,660	70.3	
	South	7,933	64.7	

Weighted prevalence of at least one of seven geriatric syndromes (cognitive, hearing, and vision impairment; falls in past 2 years; urinary incontinence; depression [CES-D 10 ≥ 4]; underweight [BMI < 18.5]) among adults aged ≥ 60 years (N = 30,811). N column shows unweighted counts; prevalence column shows weighted percentages. p-values from Pearson chi-square tests.

**Table 3:** Prevalence of individual geriatric syndromes by multimorbidity status

Geriatric syndrome	Without MM (%)	With MM (%)	Total (%)	P value
Cognitive impairment (FGCP < 10th percentile)	10.5	7.9	9.9	< 0.001
Hearing impairment	8.0	12.4	9.1	< 0.001
Vision impairment (poor distance/near eyesight)	27.3	28.5	27.6	0.498
Falls in past 2 years	21.1	25.7	22.2	< 0.001
Urinary incontinence	3.2	6.6	4.0	< 0.001
Depression (CES-D 10 ≥ 4 symptoms)	29.4	34.3	30.6	< 0.001
Underweight (BMI < 18.5)	30.4	11.8	26.1	< 0.001
Number of GS = 0	29.2	30.6	29.5	
Number of GS = 1	33.8	33.7	33.8	
Number of GS = 2	22.4	21.5	22.2	
Number of GS = ≥ 3	14.5	14.2	14.5	

Weighted prevalence of each of the seven geriatric syndromes among older adults without and with multimorbidity, and total prevalence (N = 30,811). p-values from Pearson chi-square tests. CES-D 10 = 10-item Center for Epidemiologic Studies Depression scale; BMI = body-mass index; FGCP = factor analysis general cognitive score (harmonised LASI variable r1fgcp).

**Table 4:** Life satisfaction by multimorbidity and geriatric syndrome status

Group	Mean ± SD	Low %	Medium %	High %
Multimorbidity				
No	23.41 ± 7.54	33.7	23.4	42.8
Yes	23.97 ± 7.59	31.2	22.1	46.8
Geriatric syndromes				
No	25.66 ± 7.07	22.4	21.3	56.3
Yes	22.65 ± 7.58	37.6	23.9	38.5
MM + GS co-occurrence				
No	23.65 ± 7.55	32.6	23.0	44.4
Yes	22.98 ± 7.57	35.9	23.9	40.3
4-group MM x GS				
Neither	25.49 ± 7.03	23.0	22.4	54.6
MM only	26.21 ± 7.16	20.6	17.9	61.5
GS only	22.55 ± 7.57	38.2	23.9	38.0
Both	22.98 ± 7.57	35.9	23.9	40.3

Weighted mean life satisfaction score (SWLS, range 5–35) and weighted distribution across Low (5–20), Medium (21–25), and High (26–35) categories, by multimorbidity status, geriatric syndrome status, MM × GS co-occurrence, and four-group MM × GS classification (neither; MM only; GS only; both). All estimates are weighted.

**Table 5:** Multivariable weighted linear regression estimates for life satisfaction

Variable	Model I	Model II	Model III	Model IV
Multimorbidity (ref: No)				
Yes	+ 0.56 [+ 0.32, + 0.81]***	+ 0.72 [+ 0.31, + 1.14]***		+ 0.08 [−0.33, + 0.49]
Geriatric syndromes (ref: No)				
Yes	−3.01 [−3.23, −2.80]***	−2.94 [−3.19,−2.69]***		−2.36 [−2.61,−2.11]***
MM + GS co-occurrence (ref: No)				
Yes	−0.67 [−0.95, −0.39]***	−0.29 [−0.80, + 0.21]		−0.19 [−0.69, + 0.30]
Age (ref: 60–69)				
70–79			+ 0.29 [+ 0.06, + 0.53]*	+ 0.45 [+ 0.22, + 0.68]***
≥80			+ 0.48 [+ 0.12, + 0.84]**	+ 0.84 [+ 0.48, + 1.20]***
Sex (ref: Male)				
Female			+ 0.35 [+ 0.12, + 0.58]**	+ 0.40 [+ 0.17, + 0.62]***
Living arrangement (ref: Living alone)				
With spouse only			+ 2.45 [+ 1.90, + 3.01]***	+ 2.26 [+ 1.71, + 2.81]***
With spouse and children/others			+ 3.22 [+ 2.71, + 3.74]***	+ 2.99 [+ 2.47, + 3.50]***
With children/others (no spouse)			+ 2.34 [+ 1.82, + 2.87]***	+ 2.24 [+ 1.72, + 2.76]***
Education (ref: <Upper secondary)				
Upper secondary			+ 2.26 [+ 1.97, + 2.54]***	+ 1.94 [+ 1.65, + 2.22]***
Tertiary			+ 3.45 [+ 2.93, + 3.96]***	+ 2.94 [+ 2.42, + 3.45]***
Wealth quintile (ref: Poorest)				
Poorer			+ 0.61 [+ 0.28, + 0.94]***	+ 0.58 [+ 0.25, + 0.91]***
Middle			+ 1.03 [+ 0.70, + 1.36]***	+ 0.99 [+ 0.67, + 1.32]***
Richer			+ 1.12 [+ 0.78, + 1.45]***	+ 1.04 [+ 0.71, + 1.37]***
Richest			+ 1.62 [+ 1.28, + 1.97]***	+ 1.56 [+ 1.21, + 1.91]***
Residence (ref: Rural)				
Urban			+ 0.68 [+ 0.43, + 0.92]***	+ 0.48 [+ 0.24, + 0.72]***
Region (ref: North)				
Central			+ 0.11 [−0.20, + 0.43]	+ 0.25 [−0.06, + 0.56]
East			−1.12 [−1.45, −0.80]***	−0.86 [−1.18, −0.54]***
Northeast			+ 0.81 [+ 0.43, + 1.19]***	+ 0.80 [+ 0.42, + 1.18]***
West			+ 2.63 [+ 2.23, + 3.02]***	+ 2.67 [+ 2.28, + 3.06]***
South			+ 0.18 [−0.11, + 0.47]	+ 0.11 [−0.18, + 0.40]

Estimates from weighted ordinary least-squares linear regression with sandwich-estimator (Huber–White) standard errors (N = 30,811). β coefficients represent change in SWLS score associated with each predictor. Model I: each predictor entered alone (bivariate). Model II: MM, GS, and MM × GS interaction term jointly. Model III: sociodemographic covariates only. Model IV: full adjustment for MM, GS, MM × GS, and all sociodemographic covariates.

Significance levels:

* p < 0.05;

** p < 0.01;

*** p < 0.001.

## Data Availability

The datasets used and analysed during the current study are publicly available. LASI Wave 1 data are available from the International Institute for Population Sciences at https://www.iipsindia.ac.in/lasi (accessed 8 March 2026) upon registration. The Harmonized LASI Version A.3 dataset is available from the Gateway to Global Aging Data, University of Southern California, at https://g2aging.org (accessed 8 March 2026) upon registration.

## Abbreviations

AIIMS: All India Institute of Medical Sciences
ANM: Auxiliary Nurse Midwife
ASHA: Accredited Social Health Activist
BMI: Body-Mass Index
CES-D 10: 10-item Center for Epidemiologic Studies Depression scale
CI: Confidence Interval
CIDI-SF: Composite International Diagnostic Interview – Short Form
CRediT: Contributor Roles Taxonomy
GS: Geriatric Syndrome(s)
ICMR: Indian Council of Medical Research
IIPS: International Institute for Population Sciences
LASI: Longitudinal Ageing Study in India
MM: Multimorbidity
NPHCE: National Programme for Health Care of the Elderly
OLS: Ordinary Least Squares
PHQ-2: 2-item Patient Health Questionnaire
PSU: Primary Sampling Unit
SD: Standard Deviation
STROBE: Strengthening the Reporting of Observational Studies in Epidemiology
SWLS: Satisfaction with Life Scale
